# Safety and Toxicity Implications of Multifunctional Drug Delivery Nanocarriers on Reproductive Systems *In Vitro* and *In Vivo*

**DOI:** 10.3389/ftox.2022.895667

**Published:** 2022-06-15

**Authors:** Anas Ahmad

**Affiliations:** ^1^ Department of Pharmacology, Chandigarh College of Pharmacy, Chandigarh Group of Colleges, Mohali, India; ^2^ Julia McFarlane Diabetes Research Centre and Department of Microbiology, Immunology and Infectious Diseases, Snyder Institute for Chronic Diseases and Hotchkiss Brain Institute, Cumming School of Medicine, University of Calgary, Calgary, AB, Canada

**Keywords:** reproductive toxicology, multifunctional nanoparticles, drug delivery, nanotoxicology, fetal toxicity

## Abstract

In the recent past, nanotechnological advancements in engineered nanomaterials have demonstrated diverse and versatile applications in different arenas, including bio-imaging, drug delivery, bio-sensing, detection and analysis of biological macromolecules, bio-catalysis, nanomedicine, and other biomedical applications. However, public interests and concerns in the context of human exposure to these nanomaterials and their consequential well-being may hamper the wider applicability of these nanomaterial-based platforms. Furthermore, human exposure to these nanosized and engineered particulate materials has also increased drastically in the last 2 decades due to enormous research and development and anthropocentric applications of nanoparticles. Their widespread use in nanomaterial-based industries, viz., nanomedicine, cosmetics, and consumer goods has also raised questions regarding the potential of nanotoxicity in general and reproductive nanotoxicology in particular. In this review, we have summarized diverse aspects of nanoparticle safety and their toxicological outcomes on reproduction and developmental systems. Various research databases, including PubMed and Google Scholar, were searched for the last 20 years up to the date of inception, and nano toxicological aspects of these materials on male and female reproductive systems have been described in detail. Furthermore, a discussion has also been dedicated to the placental interaction of these nanoparticles and how these can cross the blood–placental barrier and precipitate nanotoxicity in the developing offspring. Fetal abnormalities as a consequence of the administration of nanoparticles and pathophysiological deviations and aberrations in the developing fetus have also been touched upon. A section has also been dedicated to the regulatory requirements and guidelines for the testing of nanoparticles for their safety and toxicity in reproductive systems. It is anticipated that this review will incite a considerable interest in the research community functioning in the domains of pharmaceutical formulations and development in nanomedicine-based designing of therapeutic paradigms.

## Introduction

Nanomedicinal and other nanoplatform-based drug delivery systems are comparatively newer but speedily evolving paradigms, where several organic and inorganic materials lying in the nano-scale range are used as needed in disease diagnosis and for delivering the therapeutic regimen into specific target tissue localities in a well-controlled fashion. Nanotechnology-based platforms offer several profits in the treatment of chronic human disorders by site-specific and target-oriented deliverance of precision medicine. Lately, there have been a large number of appreciable purposes for which these nanomedicines (including anti-cancer chemotherapeutics, biologicals, and immunotherapeutics) are used for therapy treatment of several disorders (Patra et al., 2018). The enormous growth in these nanotechnologies along with their outstanding advantages has nurtured various fears regarding the probable health-related risks in the context of drug delivery nanoparticles (NPs) (Ema et al., 2010). These types of merchandise have currently found broad applicability in almost all fabricating sections. For example, advancements in nano-based medicines can offer a solution for earlier disease diagnoses and in individual-specific personalized nanomedicines related to the treatment of complex disorders, including tumors and several other metabolic diseases (Ventola, 2017).

Nanotechnology-based paradigms have also yielded potential platforms for providing aid in resolving social difficulties, including energy storage and conservation, as well as environmental contamination from various pollutants. Due to some of their distinct characteristic features, nanoparticles have greatly been employed in bio-medical and industry-based purposes. Currently, there have been approximately 2,000 existing consumer-based marketed commodities based on NPs, which include antibiotic formulations, food-based items, textile products, sports goods and other tools, and electronic merchandise, and furthermore, this number is ever enhancing in a continuous manner (Diallo et al., 2013; Bandala and Berli, 2019).

**FIGURE 1:**
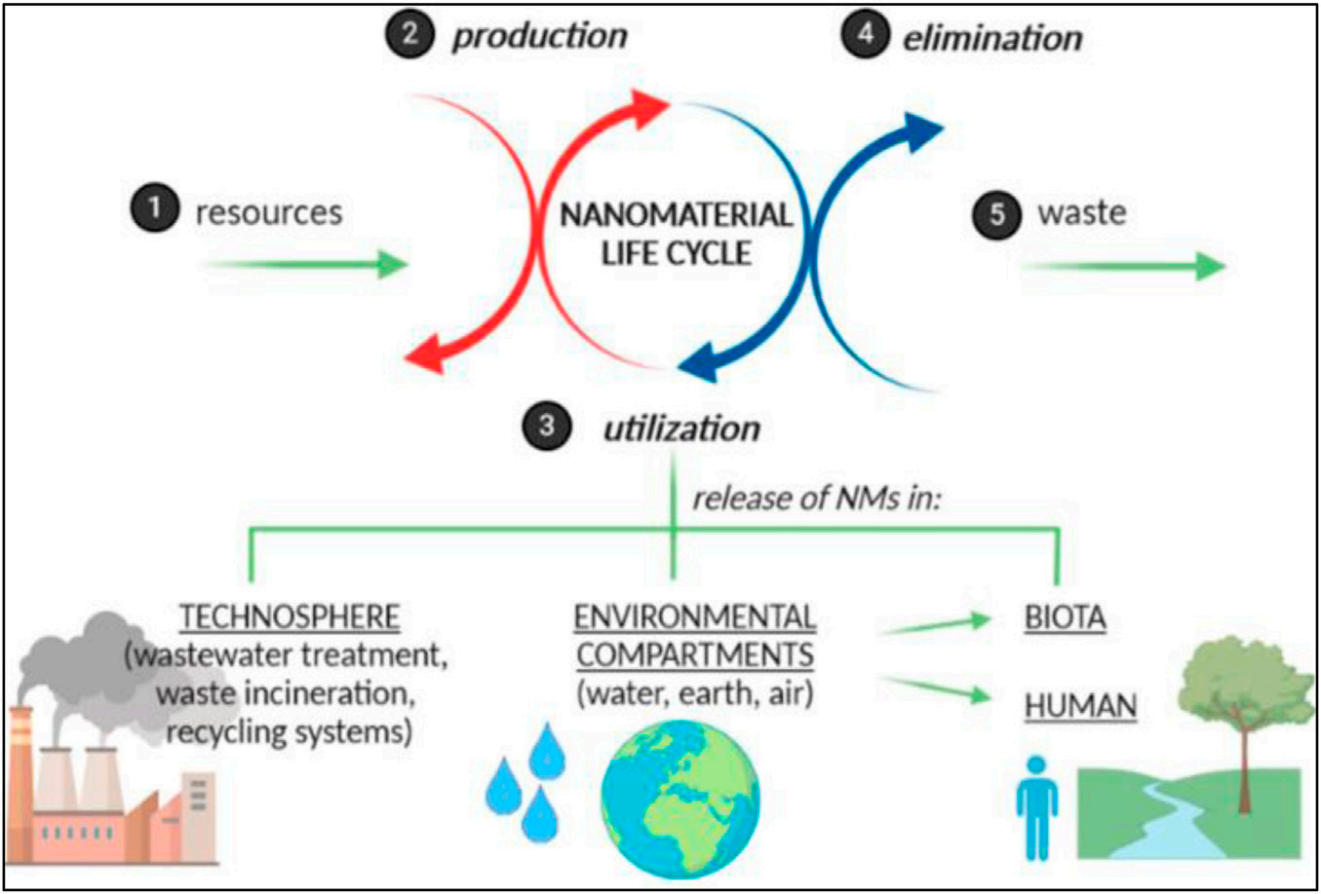
Diagrammatic representation of various effects of nanomaterials on human and environmental processes (reprinted with permission from Zielińska et al. (2020)).

Regardless of various beneficial applications of nanoparticles, several uses of these NPs could expose human beings or other animals to some of their toxicological effects. Regarding nanoparticle exposure in humans, these nanomaterials enter the human body by inhalational routes, direct oral ingestion, internalization of cellular uptake into skin tissues, *via* the parenteral injections, or through various types of implantational devices (Figure 1). It becomes interesting that nanoparticle entry can either be with a particular intention or work well without any intention. Hence, the broad applications of nanoparticles have raised various concerns and worries regarding many negative effects of nanomaterials on human health in general and reproductive health in particular, especially on the male and female reproductive tissues and on fetal developmental phenomena, specifically regarding smaller sizes of these nanoparticles, their comfortable penetrability and cytocompatibility, and the potential abilities to violate or transgress placental barriers (Bartneck et al., 2012; Yah et al., 2012; Schmidt et al., 2015). Earlier research reports on the anthropogenetic nanoparticles, viz., diesel exhausts, have exhibited that because of the daily exposure, these can accumulate and bind well to human and animal tissues, perturbing the normal systemic physiologies. Furthermore, nanoparticles have been linked to various diseases in animal systems, viz., pulmonary injuries, liver toxicities, immunotoxicity, nanotoxicity, neuronal toxicity, renal toxic consequences, and permanent testicular damages (Bartneck et al., 2012; Yah et al., 2012; Schmidt et al., 2015).

Nanomedicines could be used as some drug agents or often as drug-delivering vehicles or for the provision of fluorescent imaging-based platforms in the reproductive system. Nanoparticles employed as drug-delivering carriers for targeted therapeutics often are combined with some targeting organ or targeted cell, which can offer a chance for these nanoparticles to enter the reproductive tissues and cells. Supersensitive molecule-based imaging nano-probes could be well applied for the detection of the directed biological anatomies. For example, gold nanoparticles have proved to be dependable and label-free demarcation and contrasting medium to visualize cells of the ovarian carcinoma. In addition, other nanoparticles could be well applied for the treatment of various other disorders. The tocotrienol nanosized emulsions based on curcumin have been applied for the treatment of tumors of the breast and ovaries. Fatty acid or PLGA poly (lactic-co-glycolic acid)-based nanoparticles are applied as drug-delivering platforms for enhancing *in vitro* cytotoxic characteristics and for *in-vivo* targeting capabilities of PTX (paclitaxel) for cancer of ovarian stem cells (Lee et al., 2007; Steuber et al., 2016; Abou-ElNaga et al., 2017; Suarasan et al., 2018). All these approaches expose the reproductive system to nanoparticles and lead to direct nanoparticle entry into reproductive tissues. Furthermore, there are other indirect pathways through which nanoparticles get entry into reproductive tissues, including but not limited to absorption of nanoparticles, translocation of nanoparticles, and nanoparticle disposition. These nanoparticles can possess safety and toxicity implications in both male and female reproductive systems in terms of their potential adverse impacts on the male reproductive organs such as germ cells, sperm count, morphology of sperm cells and their dynamic motilities, and potential adverse effects on hormonal levels. In the context of the female reproductive system, these nanoparticles exert their adverse reactions on female reproductive organs, adversely impact sexual behavior, affect the follicular cells, affect ovarian and endometrial tissues, and adversely affect the hormonal levels (Wang M. et al., 2018).

## Toxic Effects of Engineered Nanoparticles on Reproduction and Development

Natality, reproducibility, and developmental paradigms of the fetus are necessities for sustaining any species, underlining the significance of arising general public cognizance regarding the nanoparticles’ toxicities on reproductive health. Women possess somewhere around few hundreds of follicles which attain a mature stage and experience ovulation in their lifetimes, signifying the quite circumscribed and confined opportunities for reproduction (Teng et al., 2021). Furthermore, the female reproductive system, which includes the ovaries and uterus, demonstrates periodical maturation, development, and regenerative phenomena which are well-governed by a specific set of hormones. This hormonal regulation possesses dynamical functionalities and becomes prone to pathophysiological strain due to foreign nanoparticles, and any such disruptions in the female reproductive system can probably culminate into fetal abnormalities (Sun et al., 2013).

Nanoparticles may cause some potential harm to the hypersensitive female reproductive system, and their toxicities have been researched in various models of female reproduction. Both acute and chronic toxicological implications in humans and animals are reported. Furthermore, various reports have demonstrated the pharmacological effects of nanoparticles on the disjunct pathophysiological systems, such as tissues and organs, biological macromolecules, and primary cell lines (Oberdörster et al., 2005). Overall, these reports have elicited several concerns, and it has become evident that much more efforts are required for determining the mechanism through which nanoparticles impact the particular reproductive tissue system. Moreover, nanoparticles could get well distributed in various reproductive tissues and organs, and their harmful effects may get carried among these reproductive tissues, hampering the entire organism. This will never be restricted to the female reproductive system. Nanoparticles can further trespass the biological placental barrier protecting the fetus and can also cross several other body barriers in humans and animals, viz., the blood–testis barriers, and can get entry into the testes (Souza et al., 2021).

Much of the research on the toxicology of nanoparticles has been carried out in animal models, viz., rats, mice, guinea pigs, hamsters, and rabbits, which are genetically close to human beings. Although these represent some of the very usual animal models, their applicability is restricted by the long-developing cycles and many ethical limitations. Furthermore, it also becomes hard to investigate their developmental stages *in utero*. Therefore, a zebrafish model has been employed for the conduction of molecular-level research for studying the developmental stages of its embryos and their developmental pathophysiology (Daviau, 1999). Herein, a detailed description of *in vitro* and *in vivo* toxicity evaluation of nanoparticles in various models, including zebrafish, rats, and mice, along with the impact of nanoparticles on reproductive tissues of both genders, will be undertaken, emphasizing the consequences of human-made nanomaterials when exposed to animals.

**FIGURE 2:**
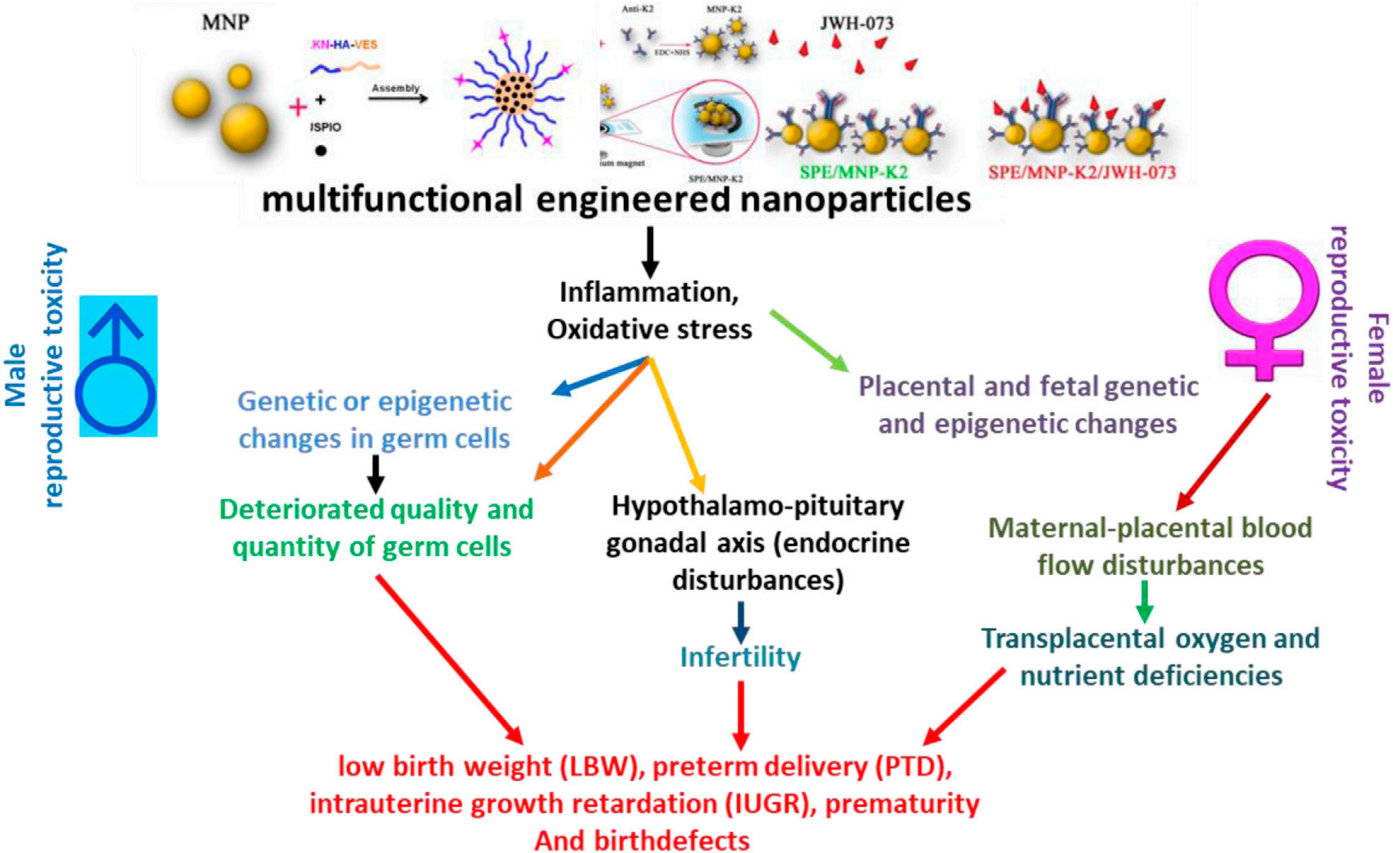
Schematic presentation of adverse impacts of engineered nanoparticles on the reproductive health of humans. IUGR, intrauterine growth retardation; LBW, low birth weight; PTD, preterm delivery. Adapted from Brohi et al. (2017) and Tran et al. (2022)

Reproductive toxicology applied to harmful and deleterious impacts on any of the stages of reproduction, pregnancy, or fetal development in animals or human reproductive periods include the interruptions in the developmental process of a healthy and sound embryo in the female gender. Adverse effects which have an impact on the developing fetus at any of the developmental stages may be the consequences of the exposure of parents to nanomaterials toxins and can be categorized as developmental toxicities. Negative impacts of various environmental contaminations, such as polluting agents, on developmental stages or the reproductive system of animals or humans are shown in Figure 2 (Brohi et al., 2017; Tran et al., 2022). Nanoparticles can also negatively impact the reproductive organs’ functions, including their pathophysiological structures, germline cellular components, and fertility paradigms.

The authors evaluated various weight-related parameters, including the maternal weight gain, placental weight, and weight of the fetus, normalized the weight of the tissues with the body weight before the statistical analyses, and concluded that the regular inhalational exposure to cadmium oxide NPs caused a delay in the mother’s weight gain, increased uterus weight, and decreased the weight of the placenta in a pregnant mouse model. Furthermore, appreciably reduced level of 17-*β* estradiol and changes in the expression level of estrogen receptors alpha and beta in uterine tissues ultimately culminated in reduced implantations. Cadmium ions, which got released from cadmium oxide NPs, could lead to endocrine disruption for the prevention of implantation and disturb and imbalance the blastocyst which could get implanted. However, the mechanism behind this phenomenon could still not be verified (Blum et al., 2012). The i. v. administration of silica and titanium oxide NPs in the concentrations of 0.8 mg in the pregnant mice demonstrated a reduction in the weight of uterus tissue and increased the rates of fetal reabsorption. These reports have well-demonstrated that NPs can adversely affect the reproductive tissues and fertilities in the female reproductive system, as could well be demonstrated by the other chemicals and toxins as well (Martino-Andrade and Chahoud, 2010; Yamashita et al., 2011). Thus, developmental toxicities occur due to trans-placental transmutability from mothers to the developing offspring. The smaller sizes of NPs and their thorough biodistribution to the reproductive system tissues and organs render them a primary candidate to cross the barrier of the placenta. To further explore these issues in depth, various placental models are being employed, which include rodents and zebrafish embryo formation model systems and perfusion model systems of the placenta from humans. These reports have confirmed that nanoparticles like those of gold, silver, titanium dioxide, silica, and carbon can easily trespass the placental barriers (Shi et al., 2013; Celá et al., 2014; Hou and Zhu, 2017).

## Nanotoxicology and Male Reproductive Systems

Several kinds of NPs have negatively impacted the germ cell lines of the male reproductive system at some or the other molecular, sub-cellular, cellular, histological, and clinical levels. Therefore, *in vitro* and *in vivo* toxicity assessments of nanoparticles in various animal models, including but not limited to rat, mouse, and zebrafish, as well as further impacts of these nanoformulations on the reproductive system of males, need to be undertaken in a detailed manner while emphasizing the outcomes of exposing these man-made nanoparticles. Several such kinds of efforts have been undertaken in the recent past (Habas et al., 2021). For example, the applications of similar types and sizes of titanium dioxide nanoparticles on the 6th, 9th, 12th, and 15th days in the 400 µg concentration in a mouse model with pregnancy conditions disclosed that in male offspring’s neurological tissues, on the 16th embryonic day striatum and on the 7th and 14th post-natal days, the olfactory bulbs and cerebral cortex tissues got enriched in the brain and major genetic changes in their expression levels in that regions and are intimately correlated with their reproductive tissues. In terms of the genetic alterations, striatum-linked genes had some differential expressional levels in the perinatal development time, while the genes linked with dopaminergic neurons and the pre-frontal cortex suffered dysregulation in the later infantile stages (Umezawa et al., 2012). Subcutaneous administration of titanium dioxide nanoparticles having a hydrodynamic diameter of less than 300 nm in the doses of 100 µg/animal during their gestational days leads to the entrance of these nanoparticles into Leydig testicles’ cellular regions, Sertoli cells, and in spermatids inside the male offspring, which were successfully observed by the electron microscopic techniques (Tang et al., 2009; Umezawa et al., 2012). The reproductive growth and maturation of male offspring can further be interrupted by nanomaterial exposure, which is composed of some of the very fine and ultra-fine nanoparticles. Nanoparticles are being used for delivering drugs such that their targeted therapies instantly get combined with the targeted organ systems or targeted cellular structures, which provide the opportunities for their entries directly into the reproductive cells and tissues. Many ultra-sensitive molecule-based bio-imaging molecular nano-probes are employed for the detection which targets the biochemistry of these nanomaterials (Borm et al., 2006; De Jong and Borm, 2008; Patra et al., 2018).

### Nanoparticle Toxicities on Seminiferous Tubules and the Process of Spermatogenesis

Nanoparticle exposure further impacts the male reproductive system cells and organs and their functions, which include the effects of the process of spermatogenesis starting from its initiation inside the seminiferous tubule in testicular tissues. In addition, nanoparticles exposing effects can further reduce spermatogenesis in terms of sperm cell production. However, this effect variegates from one species to the other. A reduction in the sperm count or yield can be directly linked to various biomolecular alterations by changing the entire genetic expression implicated in the process of spermatogenesis (Yoshida et al., 2009). A report on the effects of nanoparticles’ process of spermatogenesis gives some suggestions regarding the precautions to be taken with regards to nanoformulations in terms of the in-depth understanding of their passing on across blood–testis barriers. This issue can well initiate fetus formation when the nanoparticles get passed on *in utero*. For example, it is reported that s. c. injection of titanium dioxide nanoparticles to the mouse in its pregnancy state can lead to nanoparticles passing onto the Sertoli cellular compartments and into the spermatids inside the seminiferous tubule and their neighboring Leydig cellular structures, in male offspring. It then leads to a reduction in the counts of Sertoli cells, induces changes in testicle morphologies, and disrupted and disjointed seminiferous tubular structures. Sperm motilities in the epididymis can also get changed inside male counterparts. Thus, exposing the mice’s fetal cells and tissues to titanium dioxide nanoparticles spoils the formation, maturation, and functioning of male reproduction and regeneration systems at very primary levels with regard to the process of spermatogenesis. Likewise, intra-tracheal disposal of carbon-based nanoparticles into the mice during the pregnancy stages culminates in histopathological alterations in seminiferous tubules and decreases the daily sperm production in the male counterparts (Yoshida et al., 2010).

Alteration in seminiferous tubular morphologies and the process of spermatogenesis gets induced with the exposure of the mice to nanoparticles. Intra-tracheal disposal of carbon-based nanomaterials in higher doses and several times each week leads to histopathological alterations in the seminiferous tubule and increases the testosterone in the serum after nanoparticle exposure. The smaller sized nanoparticles have less harmful effects than the large sized nanoparticles, despite the same nanoparticle number, indicating that the hydrodynamic diameter of nanoparticles becomes a very crucial point while determining the effects of these on the process of spermatogenesis (Iftikhar et al., 2021). In another report, an i. v. administration of multi-walled carbon nanotubes inside the mouse model can induce the reversible damages in the testes but does not affect their fertilities. The thickness of the epithelia of the seminiferous tissues gets decreased and induction of severe oxidative stress takes place. This increased reactive oxygen species concentrations and thickness of the epithelia of seminiferous tissues get restored in a couple of weeks. No changes could be observed in numbers or qualities of sperms or in hormonal concentrations in mice administered with multi-walled carbon nanotubes in the prior periods, and mating among these treated mice showed normalized pregnancy conditions and quite normal and successful deliveries, demonstrating that it had no effects on their fertilities (Bai et al., 2010). These reports underline the distinctive effects of various kinds of nanoparticles and various differences among different carbon-derived nanomaterials, viz., carbon-based nanoparticles or multi-walled carbon nanotubes.

### Toxicological Implications of Nanoparticles on Testes

Nanomaterial accumulation in testicular tissues has been well-exhibited in various animal model systems, but a deep comprehension of biodistribution of nanoparticles in testicles is still restricted, and their nano-toxicological effects are hard to classify and elucidate depending solely on evidence generated from various past reports. The i. v. (intravenous) or an i. p. (intraperitoneal) injection of either sodium chloride- or iodide-coated silica nanoparticles in the concentrations of 225 mg/kg body weight into the mouse model causes the nanoparticle accumulation in testicular tissues, glands, and interstitial cellular compartments, 96 h post injection schedules. However, no further readings of any other pathophysiological cellular alterations or increased mouse mortalities could be observed (Chen et al., 2003; Brandelli, 2020). Likewise, the i. p. injection of magnetized nanoparticles coated with silica and encapsulating rhodamine-B isothiocyanate with a hydrodynamic diameter of 50 nm in various doses in male mice showed accumulation of nanoparticles in testicular tissues and without any evident toxicological outcomes. Furthermore, the delivery of fluorescence-exhibiting nanoparticles in the concentration of 1 mg/L to fish eggs exhibited that nanoparticles could be well detected in their testicular tissues (Leclerc et al., 2015). Again, no pathophysiological alterations could be evidenced inside their cellular or nuclear compartments, and no acute toxicities of nanoparticles in terms of the increased mortalities even with higher dosing could be seen. Contrastingly, another report on the i. m. administration of silica and gold nanoparticles in the mouse demonstrated no accumulative evidences of these nanoparticles of around 65–75 nm diameter in the testicles even 40–50 days post injection schedules (Ong et al., 2016).

Other kinds of nanomaterials, including but not limited to silver nanoparticles, titanium dioxide nanoparticles, and gold nanoparticles, can show testicular toxicological profiles once the testicles are exposed to such nanoparticles, and these nanoparticles get accumulated inside these tissues. These particles are found to affect a number of male reproductive aspects and functions such as germ cells’ numbers, health of tissues and cells in the male reproductive system, changes in the testicular histopathology, and sperm morphologies and motilities. Various factors such as the solubility of nanomaterials and their penetrating capabilities into testicular tissue barriers govern the toxicities of these nanomaterials in the male reproductive system (Hong et al., 2016; Lafuente et al., 2016). Water-soluble nanoparticles can easily be eliminated from the body and hence offer lesser toxicological effects than fat-soluble nanoparticles, which show preferential affinity and accumulation in tissues and hence exert higher toxicities. These nanoparticles also reduce the fertility profiles in males, induce apoptotic cell death, or sometimes necrosis of spermatogenic or Sertoli cells in higher doses, induce inflammation in the testicular or male reproductive tissues, and lead to reduced and compromised immunities in the male models (Sharma et al., 2012). Hence, it becomes necessary to carry out extensive toxicity studies for the definite evaluations of the accumulative potential of nanoparticles in various suitable animal models in the context of the male reproductive system.

### Nanoparticle Toxicities in Sperm or Germline Cells

These probable toxicities of nanoparticles inside reproductive organs and tissues can be readily assessed by analysis of the germline cellular components. Their outcomes are dependent upon various kinds of nanoparticles employed. In a report, gold nanoparticles were incorporated into freshly obtained semen samples from a healthy volunteer for investigating their implications on the health of the human sperms. These nanoparticles get accumulated in spermatozoid tail segments and head portions and lead to their non-motility in a quarter of the sample cells. It was not clear whether this immediate and intimate exposure of these cells to the nanoparticles can occur directly, although these applications of gold nanoparticles in various techniques like bio-imaging can culminate into rather higher dosing and that can ultimately impact the health of the sperms upon bio-distribution of these nanoparticles throughout the entire cells and organs (Wiwanitkit et al., 2009; Tirumala et al., 2021). Furthermore, the effects of direct mixing of the PVA (polyvinyl alcohol)-coated iron oxide nanoparticles with the sperm samples obtained from bovine were analyzed. When nanoparticles are reported to be entering the spermatozoid cells and then could get attached to their mitochondrial organelles inside their tails and also in their acrosomal regions inside their heads, there have been no direct impacts on spermatozoid acrosomal reactions and motilities (Vassal et al., 2021).

Sperm cell samples from mice can suitably fit into the *in vitro* model systems to compare the nanotoxicological profiles of various nanoparticles inside the male reproductive system research. The C4 and 18 cellular systems, obtained from the type-A spermatogonial cells or isolated from the male mice and sperm cell samples from the bovine animals, were employed for testing the abilities of polyvinyl alcohol-coated magnetite nanoparticles for entering into spermatogonial or primary cell samples without perturbing their abilities for undergoing the acrosome-based reaction or continue to be motile (Jamsai and O’Bryan, 2011). Mitochondrial functions could be well evaluated for the reading out of nanoparticles’ cytotoxicities, along with cellular morphologies, membranous leakages, and apoptotic cell deaths post-treatment and after the permeabilization of cellular membranes and releasing off the bonded nanoparticles. Significantly, nanoparticle toxicities are dependent upon particles’ doses reaching the testicular tissues, while solubilities of the salt forms did not exert any of the significant beneficial effects. Silver nanoparticles among all represent the most toxic nanomaterials; this is because their EC_50_ values in the context of the mitochondria functional assays are appreciably lower than those of the molybdenum trioxide nanoparticles. Therefore, such research reports, which can directly permit the comparison of adverse or harmful effects of various kinds of nanoparticles under similar experimental circumstances, can greatly aid in the clearing-up of confusion around the comparative impacts of various nanomaterials and their doses on male reproductive functionalities and several other arenas of reproductive system tissues (Mohammadinejad et al., 2018; Wang R. et al., 2018).

There have been various industry-based set-ups which release smaller nanoparticulate materials similar to drug delivery nanoformulations. Air-based pollution due to nanoparticulate materials and their adverse effects on animal, environmental, and human health has been the continuously evolving arenas so as to explore in the coming times (Sonwani et al., 2021). In some reports, for evaluating the adverse impacts of air-borne particles on the living systems, a mouse model was employed and exposed to closed air-flows in contaminated industrial areas nearing the steel-based manufacturing mills, and it resulted in germ-line cellular mutational changes. These results were quite considerably alarming and required many in-depth analyses aiming to evaluate the health and well-being of populations residing in peripheral regions of these industrial set-ups (Jaishankar et al., 2014).

## Nanotoxicology and the Female Reproductive System

The reports on toxicological implications of nanoparticles in female reproductive organs and tissues primarily include their actions on the reproduction abilities, teratogenicity arising through the entire process of fetal developmental stages, and effects on offsprings during their prenatal periods. In the recent past, reports have showed that the inhalational, ingestional, or dermatologically absorbed nanoparticles can get translocated with circulatory functions and can further get accumulated into various reproductive and fetal tissues. Furthermore, these reproductive toxicities of nanoparticles in distinct germline cellular locations *in vitro* and animals *in vivo* have been progressively accounted for. For example, *in vitro* research reports have shown that few nanoparticle types can get engulfed by the granulosa cells, leading to alterations in hormonal secretions and abnormalities of ova (Hougaard et al., 2015; Dugershaw et al., 2020). There have been quite well-established research reports exhibiting that nanoparticles could get entry into both the thecal and/or granular cell compartments and impact the normal functionality, especially in the context of their vital roles in hormonal secretions. Before the process of ovulation, androgen hormones and androstenedione are released by the thecal cell structures which then diffuse into granular cells and get converted into steroidal hormonal moieties. During these procedures, nanoparticles can immediately impact the secretions of sexual hormones by disrupting the functions of these secretory cellular structures inside ovarian tissue. *In vivo* reports showed that chronic administration of TiO_2_ (titanium dioxide) nanoparticles in the female mouse culminates in the misbalancing of sexual hormones and mineral elemental bio-distribution, leads to a decrease in their pregnancy rates and reactive oxygen species production, and disrupts the ovarian genetic expressions. Further, *in vivo* experimentations in rat models demonstrated that silver nanoparticles can get transported from maternal tissues to the offspring *via* the placental routes or sometimes through the breast milk as well (Das et al., 2016; Chen et al., 2017).

### Toxicity of Nanoparticles on Female Reproductive Organs

Nanoparticles adversely impact the uterine and ovarian tissues by the subaltern disposition just after the blood has been circulated through these tissue systems and organs. Reports have shown that small-sized nanoparticles have a better ability to accumulate in uterine tissues. For example, exposing the zinc oxide nanoparticles either prior to or during pregnancy states and during the lactation periods can appreciably offer various health-related dangers to pregnant ladies and their developing embryos (Hou and Zhu, 2017; Wang M. et al., 2018). One such report observed that nanocapsules or other nano-seized lipid-based emulsion agents and also nanoparticles get accumulated in specified localizations in several rodent ovarian tissues. This process could be further assessed by the *ex vivo* fluorescent bio-imaging and confocal microscopic techniques. On the basis of this extensive *in vivo* and *ex vivo* research, these implications of nanoparticle toxicities were found to be size-dependent (Anton et al., 2008; Clementino et al., 2021).

In these contexts, large-sized nanoparticles are reported to be accumulating more than the small-sized particles. Since these accumulation patterns are limited to some of the very specific localizations inside the ovarian tissues, the toxicological risks for humans have rather been reduced or minimal. Few research studies further suggested that nanoparticles can get deposited inside the ovarian tissues and adversely impact the reproductive organ systems (Schädlich et al., 2012; Jo et al., 2013; Kong et al., 2014; Raj et al., 2017). One such study showed that the reduction in the ovarian weights and its coefficient was due to the toxicity of nickel-based nanoparticles. Short-termed (acute) as well as long-term (chronic) exposing the tissues of *Drosophila* to silver nanoparticles during their adult stages can considerably impact the egg-laying capabilities and thus can result in disturbed ovarian development and growths (Schädlich et al., 2012; Jo et al., 2013; Kong et al., 2014; Raj et al., 2017) (Schädlich et al., 2012; Raj et al., 2017).

### Toxicological Impacts of Nanoparticles on Oogenesis

Toxicological implications of nanoparticles variegate with their shapes, hydrodynamic diameters, particle sizes, surface charges, surface coatings, other constituent materials, concentrations and doses, routes of their and extent of exposure times, and tolerability in various cells (*in vitro* models) and animals *in vivo*. Various studies on toxicological outcomes of nanomaterials in reproductive cellular systems and tissues of females majorly implicate metals and/or metallic oxides, carbon-based nanoparticles, and cadmium and/or selenium core–based quantum dots (see Table 1 for details of all these types of nanoparticles) (Yin et al., 2005). *In vivo* and *in vitro* results demonstrated that various size ranges of nanoparticles can well penetrate into the various female germline cellular compartments and get accumulated inside those, which can then start the various cellular reactions including but not limited to the production of reactive oxygen species, DNA damage, apoptotic cell deaths, inflammatory responses, and inhibiting the various signal transduction mechanisms (Mao et al., 2022).

**TABLE 1:** Adverse effects and toxicological implications of various types of nanoparticles in the female reproductive system and stages of fetal development.

Type of NPs	Size/size range	Dose/concentration	Animals/model	Toxicological outcomes	Reference
Polyvinyl pyrrolidone–coated silver NPs	20–50 nm	0.427, 0.407, and 0.013 mg/kg	Rats	NPs are causing impairing of cognition in the offspring.	Wu et al. (2015)
Gold NPs	10	2.85 × 10^10^ NPs/ml	*In vitro*	NPs are found to be affecting steroidogenetic capacities by the granulosa cells in culture media after trespassing through the granulosa cell membranes.	Lyngdoh et al. (2020)
Silver nanoparticles	14 nm	50 nM	Mice	Inner cellular mass was subjected to the induced apoptosis, and embryonic growth shows trophectoderm.	Li et al. (2010)
Gold NPs	13	0.9–7.2 μg/g body weight	Mice	NPs get accumulated in the placental and fetal tissues.	Yang et al. (2018); Bongaerts et al. (2020)
Silver nanoparticles	35 nm	1.69–2.21 mg/kg	Rats	NPs show appearance in the fetal growth.	Melnik et al. (2013)
Gold NPs	20	1 nm for 48 or 72 h	*In-vitro*	NPs cause the alteration of almost 19 genetic makeups in the fibroblast cells of the lungs of the fetus.	Gedda et al. (2019)
Silver nanoparticles	8 nm	250 mg/kg	Rats	Pups’ tissues exhibit NP accumulation.	Lee et al. (2012)
Gold NPs	20 & 50	0.01%	Mice	NPs can travel through the placenta through endocytic vesicular transportation	Rattanapinyopituk et al. (2014)
Silver nanoparticles	–	0.001–100 μg/ml	*In vitro*	NPs show interference in the reproductive tissue function and alter levels of E2 and P4.	Scsukova et al. (2013)
Gold NPs	3, 13 & 32 nm	0.9 μg/g body weight	Mice	NPs are found to be enhancing the inflammation of uterine tissues and get accumulated in fetal tissues	Tian et al. (2013)
Silver nanoparticles	–	0.09–1.0 mg/ml	*In vitro*	Intervention by NPs in proliferative pathways and cause apoptotic implications in granulosa cell lines of pork ovaries	Kolesarova et al. (2011)
Gold, silver, and gold-silver alloy	6 and 20 nm	0.66 g/L for alloy, 2.5 g/L for silver, and 0.5 g/L for gold	Pigs’ *in vitro* ovaries	NPs are found to be inhibiting the maturation of oocytes, and toxic impacts are increased by the NPs of alloys.	Tiedemann et al. (2014)
Silver nanoparticles	35 nm	1.69–2.21 mg/kg	Mice	NPs travel in the mother’s breast milk and get accumulated in the developing embryos	Melnik et al. (2013)
Titanium dioxide NPs	5.5 nm	10 mg/kg	Mice	Initiation of premature oogenesis and causing the apoptotic cell death in ovarian cells, enhancing the atresia in primary and secondary follicular developmental stages	Zhao et al. (2013)
Silver nanoparticles	55 nm	0.2–20 mg/kg	Rats	Nanoparticle demons	Charehsaz et al. (2016)
Titanium dioxide NPs	25 nm	--	*In vitro*	Deformation of follicular growth and inhibition of the maturation of oocytes	Hou and Zhu, (2017)
Silver nitrate NPs	55 nm	20 mg/kg	Rats	NPs damage neurons in the hippocampal regions of the brains of both adults and offspring.	Charehsaz et al. (2016)
Titanium dioxide NPs	13–27 nm	1–5 μg/ml	Chinese hamster ovary cell line	Genotoxic and cytotoxic outcomes	Kazimirova et al. (2020)
Cadmium oxide NPs	11–15 nm	100 or 230 µg	Mice	Placental toxic reactions	Blum et al. (2012)
Silver or silver nitrate NPs	10 nm	66 mg/kg	Mice	NPs caused hampering of the growth of embryos	Austin et al. (2016)
Titanium dioxide NPs	--	2.5, 5, and 10 mg/kg body weight	Mice	Alteration in the expressions of relevant ovarian genes in a concentration-dependent manner	Zhao et al. (2013)
Titanium dioxide NPs	--	0.001–100 μg/ml	*In vitro*	Alteration in the levels of P4 and E2 and interference in reproductive system functions	Karimipour et al. (2018)
Titanium dioxide NPs	50 nm	1 μg/ml	Mice	NPs crossed the placental barrier and hampered the central nervous system development in the fetus	Shimizu et al. (2009); Umezawa et al. (2012)
Titanium dioxide NPs	10 nm	100 mg/kg body weight	Rats	NPs exerted neurotoxicity in the brains of neonates and adults	Mohammadipour et al. (2014), Mohammadipour et al. (2016); Ebrahimzadeh Bideskan et al. (2017)
Titanium dioxide NPs	∼100 nm	100 mg/kg body weight	Rats	Induction of apoptotic phenomena and reduction in neurogenesis	Mohammadipour et al. (2014), Mohammadipour et al. (2016); Ebrahimzadeh Bideskan et al. (2017)
Titanium dioxide NPs	35 nm	0.8 mg per animal	Mice	Accumulation of NPs in the brain, placental trophoblasts, and liver of the fetus	Yamashita et al. (2011)
Titanium dioxide NPs	4 nm	88–108 m^2^/gm	*Ex-vivo*	Placental toxicities	Blum et al. (2012)
Aluminum oxide NPs	9–47 nm	1–25 μg/ml	Chinese hamster ovary cell line	Cytotoxic and genotoxic effects	Di Virgilio et al. (2010)
Cerium oxide NPs	35 nm	100 µm	Mice	Adverse reactions on oocytes	Courbiere et al. (2013)
Cerium oxide NPs	35 nm	10 & 100 µm	Mice	NPs got aggregated and accumulated in follicular cells by the endocytotic mechanism and showed distribution in zona pellucida of oocyte cells	Preaubert et al. (2016)
Zinc oxide NPs	∼100 nm	500 mg/kg	Rats	Reduction in the numbers of live-born pups and enhancement of fetal repsorptive phenomena	Jo et al. (2013)
Zinc oxide NPs	∼20 nm	50 or 100 mg/kg	Hens	Inflammatory responses, ROS production, and disturbances in the signaling pathway	Liu et al. (2016)
Polyethylene imine and PAA-coated iron oxide NPs	28–30 nm	50 mg/kg body weight	Mice	NPs lead to the death of the fetus.	Di Bona et al. (2014)
Alpha-iron oxide NPs	50 & 70 nm	100 μg/ml	*In vitro*	Oxidative stress and cellular death	Faust et al. (2014)
Cadmium oxide NPs	11–15 nm	100 and 230 mg/m^3^	Mice	NPs show accumulation in placental tissues and an increase in the weight of the fetus.	Liu et al. (2017)
Silver NPs	5–70 nm	0.2 & 2 mg/kg	Mice	NPs caused neurobehavioral impairments in the offspring.	Ghaderi et al. (2015)
Copper oxide NPs	4 nm	40–44 m^2^/g	*Ex vivo*	Reduced cell viabilities and reduction in levels of human chorionic gonadotropins	Blum et al. (2012)

Some recent research reports have exhibited some of the newer phenomena where mammalian oocyte has displayed various toxicological reactions to Au (gold) and Ag (silver) nanoparticles, as well as other nanoparticles based on their alloys. These reports have shown that both Ag and Au nanoparticles majorly get accumulated in the cumulus cellular layer and in oocyte cells. In addition to that, reports have yielded various evidence that toxicological outcomes to these oocyte cells get increased with Ag molar fractions. Other *in vitro* models of zebrafish ovary follicular cells around the oocytes, thecal cellular regions, and granulosal cell localities exhibited the apoptotic cell death having the phenotype of irregular cellular morphologies, unorganized cytoplasmic regions that are disunited and fractionated, and condensed nuclear regions post-exposure to silver nanoparticles and silver nitrate (Ferdous and Nemmar, 2020; Dhanker et al., 2021). Various *in vitro* experimentations have also demonstrated that quantum dots conjugated with transferrin molecules can be chiefly uptaken by cumulus cellular structures; however, no quantum dot was able to get entry into the oocyte cells. With the continuously enhancing concentrations of conjugates of quantum dots with the transferrin molecules, the formation of cavities could get retarded and *in vitro* rates of oocyte cellular maturation could well exhibit an apparent downregulation (which was reported to be lowering from 62% to 22%). The authors have concluded that both quantum dots and conjugates of quantum dots and transferrin molecules can well demonstrate cytotoxic reactions. They resolved that these mechanisms of interfering with oogenesis processes could be well mediated through the disturbances in these cavity formations in the oocyte cells, dysfunctioning the other cumulus cellular regions and disturbances in the signals’ transduction mechanisms between the germline cellular regions and somatic cells (Prasad et al., 2010; Kapur et al., 2018).

As detailed in the previous discussion, most of the ovary follicular cells experience abnormalities while being in their developmental stages, which sometimes become a procedure implicating the hormone-regulated well-controlled apoptotic process governed by many of the factors. With prolonged exposure of these cellular structures to titanium dioxide nanoparticles, the expressional level of approximately 290 genes implicated in hormonal and cytokine metabolic processes could be found to be changing in the mouse ovarian cells. Authors have also described that these inflammatory conditions of ovaries and follicles’ abnormalcy could be because of the titanium dioxide nanoparticles in dose-dependent function, changing the expressional level of various genes in ovarian follicles (Chen et al., 2017; Gifford et al., 2017; Hou and Zhu, 2017). *In vivo* studies in female rat models when exposed to nickel nanoparticles of hydrodynamic diameter of 90 nm in the dose of 15 or 45 mg/kg showed toxicities of reproductive tissues, viz., swellings of mitochondria, cristae of mitochondria disappearing, and enlargements in their endoplasmic reticulum of ovarian follicles. Because of the appreciable lowering in the activities of SOD and catalase enzymes and increased production of reactive oxygen species, malondialdehyde and nitric oxide, and increased expressional level of apoptotic favoring protein molecules such as cytochrome-C, Bid, Bax, and Fas, the probable damaging mechanisms can be well observed because of the ROS (reactive oxygen species) productions and apoptotic processes accelerated by nickel nanoparticles (Kong et al., 2014; Abdulqadir and Aziz, 2019).

### Adverse Impacts of Nanoparticles on Female Hormonal Systems

The hypothalamus pituitary ovarian axis plays an important role in the adjustment and regulation of secreting several neuro-hormonal mediators, viz., gonadotropin-releasing hormone, luteinizing hormone, and follicle-stimulating hormones which control the reproductive organs and tissues. Nanoparticles when exposed through inhalational routes could well travel across or surpass the blood–brain barriers through the circulatory system and then further get accumulated inside the central nervous system. These exposures of nanoparticles in females can hamper the normal functioning of the hypothalamus pituitary ovarian axis and therefore can increase the risks associated with the neuro-hormonal instabilities (Kong et al., 2016; Hou and Zhu, 2017). An *in vivo* report details about the female rat model, when exposed to nickel nanoparticles, can demonstrate sexual hormonal imbalances (for example, increment in follicle-stimulating hormones and luteinizing hormones and lowering down of the estradiols) and also cause damage to the ovarian tissues, including the enhancement of cellular apoptosis, infiltrating inflammatory cells, lymphocytosis, or sometimes vascular dilation. Female rat models from their post-natal days were routinely administered with the i. p. injection of PEG-block-PLA nanoparticles in fixed doses. The outcomes exhibited that neonatal rats administered with these nanoparticles had highly afflicted reproductive tissue growth along with the disturbed releasing of the luteinizing hormones by their pituitary glands, finally culminating in the reproductive malfunctioning once they attain the maturity (Scsukova et al., 2015).

Exposure to titanium dioxide nanoparticles can appreciably change the level of sexual hormonal mediators, which include luteinizing hormones, follicle-stimulating hormones, progesterones, and estradiol and result in the increased abnormalities of primary and secondary follicular developmental processes and decreased natality. Granulosa cellular regions of the ovaries get further implicated in steroid genesis and are significant for regulating ovary functions. *In vitro* reports demonstrated that Au nanoparticles can well cross the granulosa cellular membrane and some intra-cellular compartments as well, like the lipid droplets and mitochondrial membranes. These Au nanoparticles can adversely impact the steroid-generating capacities of granulosa cells in the culture medium. Post an incubation period, the estradiol 17-beta concentrations, released by granulosal cells, also showed considerable changes in comparison to the normal concentrations. These factors can be controlled by steroids generating enzymes side chain cleaving, localized on inner mitochondrial membranes (Jamnongjit and Hammes, 2006; Karimipour et al., 2018). These studies are consistent with other reports which exhibit that quantum dots and their conjugated counterparts can further permeate into initial protective layers of follicular cells and get accumulated inside cytoplasmic regions of thecal cellular regions and granulosal cells. This can further change the activities of the inner membranes of the mitochondria and its various enzymes for disturbing the steroid synthesis pathway. Nanoparticles formulated from calcium phosphate are some of the majorly employed carriers in bone-repairing arenas. Because of the smaller hydrodynamic diameter and distinct physiochemical features, these nanoparticles can get entry into granulosal cells in the artificial culture media and get disseminated throughout the entire membranous compartment, including lysosomal localities, mitochondrial regions, and intra-cellular vesicular localizations. Aggregation of these calcium phosphate nanoparticles could exhibit the perturbation of cell cycles in the cultured granulosa cells of human ovaries ultimately culminating in increased cellular apoptotic deaths (Wang et al., 2006; Liu et al., 2010).

## Placental Interaction of Nanoparticles and Crossing of the Blood–Placental Barrier

In recent years, the safety and toxicological implications of NPs have drawn considerable attention as the similar characteristic features which render the NPs practically applicable for their theranostics and biomedical uses and can further turn into their safety relevancies. Various *in vitro* reports claimed the direct exposure of cells inside the culture medium to NPs can result in varying degrees of cellular damaging effects and sometimes cell lethality. Furthermore, results of indirect exposure of cells to these nanocarriers, however, have not recently been studied in great detail. It, however, has also become enticing to presume that if NPs are unable in crossing some biological barriers (like the blood–brain barrier or the blood–placental barrier), organs and cellular structures across these barriers will remain spared or unharmed from the toxicological implications of nanoparticles. Various researchers have tried to explore whether these kinds of physiological and tissue barriers are able to avail protection to these tissues from various kinds of NPs (which get generated as a result of various biological biomedical processes (Bhabra et al., 2009; Myllynen, 2009). Cells from the fibroblast origin (representing the most general kinds of collagen making cellular compartments) have been grown and got matured in culture plates; some contrived cellular barriers were administered across the cellular compartments. NP delivery was then undertaken in the proximity of this barrier for representing the exposures indirectly. This barrier was consisting of “BeWo” cells derived from chondro-carcinoma (which represents the accomplished *in-vitro* placental models) and grows on the membranes in several layer-based fashions for preventing the NPs from leaking across this barrier. The design could ensure the study of only the indirect implications of NPs. Although this BeWo cellular multiple-layered model does not exactly represent the accurate replication of human placental functioning barriers, it could well represent an accomplished model system for investigating the transferring process of several molecular and chemical moieties through the placental barrier (Lewinski et al., 2008; Myllynen, 2009; Saunders, 2009).

Our interpretation of the advantageous and damaging physiological consequences of NPs continues to be uncompleted. Indeed, the various reports by researchers warn that assessments of toxicity and safety of NPs could not solely rely upon their capabilities of crossing the various protective barriers, viz., blood or placenta, directly or indirectly, rather these burdens need to be covered with adequate parallel significance. Although these models are quite largely simple and more in-depth research is required for confirming these outcomes, this becomes one of the vital lessons to be learned. Although, organs and tissues of g. i.t, placenta, skin, and lungs represent major barriers for most of the nanoparticles, there have been various albeit contradictions in the evidence that NPs from the extraneous exposures can well get translocated to several other systemic localities (Myllynen et al., 2008; Bhabra et al., 2009). For example, in the case of BeWo cells, they were raised in the form of monolayers on trans-well inserts and served as the models for placental transportation of various biochemical and pharmacological agents, viz., viruses, fatty acids, immunoglobulin, amino acids, hormones, etc. These BeWo cell-based barriers, comprising 3–4 cell-thick layers, were not meant to be the replica of the human placental barrier. However, it was aimed for creation of multilayered barriers which were deprived of any gap (which could be created by toxicological implications of metallic NPs) such that collateral exposure of NPs get tested with assurances (Bode et al., 2006; Heaton et al., 2008; Blundell et al., 2016).

**FIGURE 3:**
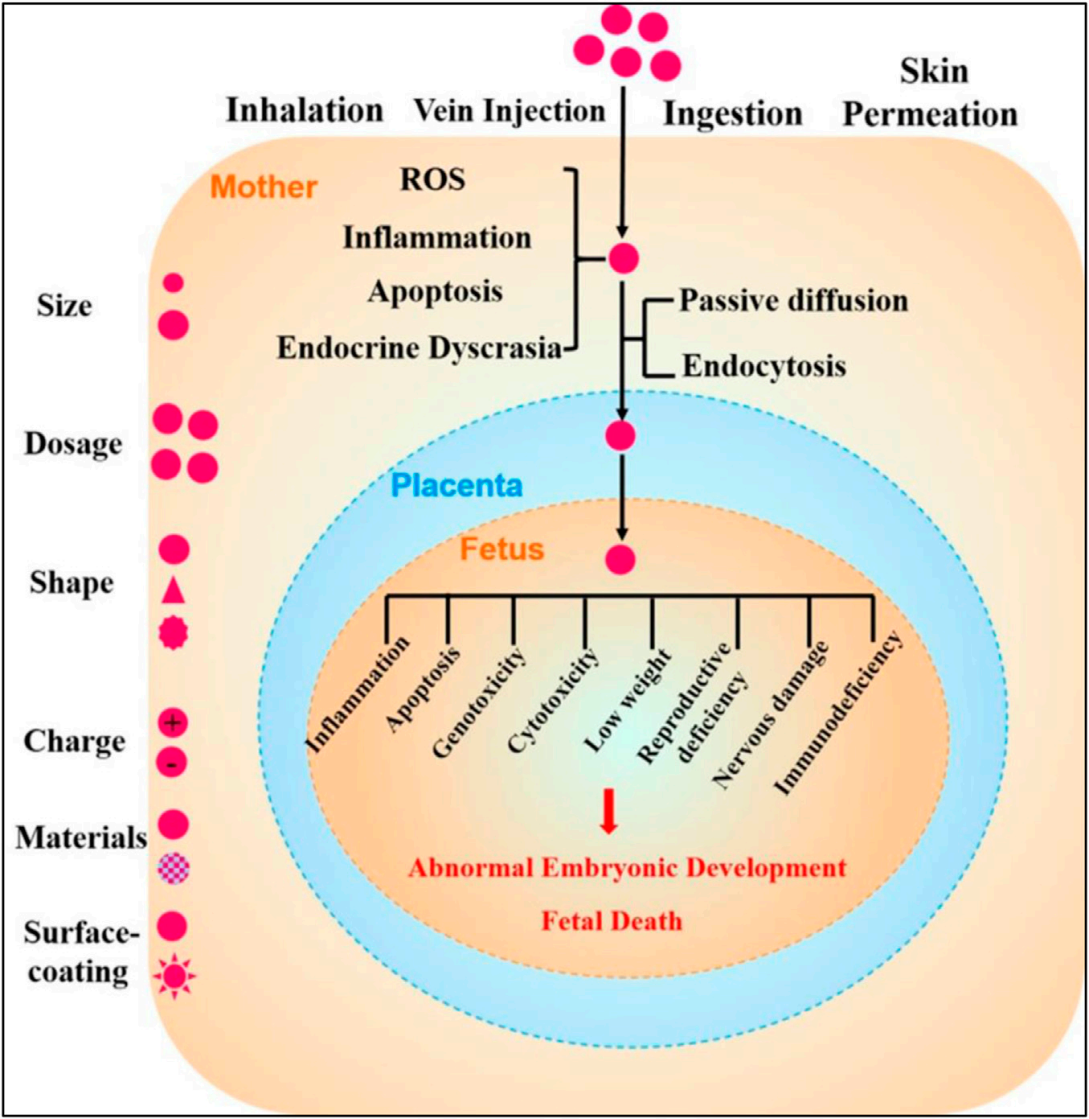
Schematic representation of the toxicological effects of nanoparticles in pregnancies and fetal developmental stages. Various factors affect the toxicities of nanoparticles which include their shapes, sizes, dosage formulations, shape, surface charge distributions, nanomaterial types, and various surface coating. Once the nanoparticles get entry into the pregnant woman’s body *via* inhalation, injections, ingestion, or skin penetrations, maternal toxicity stresses including but not limited to reactive oxygen species inflammatory conditions, and apoptotic cell death and endocrinal dyscrasias get induced. During these pregnancy conditions, nanoparticles then may cross placental barriers and can get diffused into fetal tissues by the passive diffusion mechanisms or through endocytotic pathways. These toxicological outcomes of nanoparticles may further initiate inflammation in fetal tissues, apoptotic processes, genotoxicological reactions, cytotoxicities, lower body weights, reproductional deficiencies, neuronal damages, and immunodeficiencies, among various other processes that may culminate in abnormal fetal developmental processes or sometimes fetal death (reprinted with permission from (Hou and Zhu, 2017).

Nanoparticles exert their various toxicological outcomes in both male and female reproductive tissues, but further, also exhibit their sex-dependent variabilities in the therapeutic effectiveness. Fetal hypoxic conditions which often occur during pregnancies can lead to the induction of oxidative and nitrosative stresses, and increase the risks of extremely endangering diseases at later matured stages of the matures offspring. The placenta of the male and female fetus uses distinct phenomena for suppressing these stresses and demonstrated distinct response for similar stimulus. Nanoparticles containing the MtiQ, a mitochondrial oxidation stress-suppressing antioxidants, were employed for treatment of fetal hypoxic conditions and prevention of the late-staged placental dysfunctioning in animals, demonstrating the sex-specified therapeutic outcomes favoring the female fetal counterparts. Therefore, sex differentiations need to be taken into consideration for the entire developmental stages of nanoparticle based drug delivery systems for treating the placenta related or embryological conditions (Schoots et al., 2018; Ganguly et al., 2019; Hajipour et al., 2021). Various placental and fetal toxicological implications of nanoparticles have been depicted in Figure 3.

The applications of nanoparticle-based medicines have been much enhanced, and it has become vital to comprehend their direct and indirect therapeutic and toxicological outcomes. It has also been demonstrated that the NP use could result in DNA damage inside cells which were cultured without any of the cellular barriers and the damage was seen by NPs without the barrier crossing. It was also exhibited that these DNA damages were relying on the cellular barrier’s thicknesses, and could well be arbitrated by the signaling pathways *via* gap junction’s protein after the free radicals were generated in the mitochondria (De Jong and Borm, 2008; McMillan et al., 2011). Indirect damages were also observed around both of the trophoblastic barriers and barrier of the cornea (the blood–corneal barrier). These signaling pathways, which also include the releasing of cytokines, happen only through the two-layered or multi-layered barriers, but not through the mono-layered barriers. Indirect toxicities were also seen in the mouse model and applications of *ex vivo* explants of placental tissues from humans (mimicking the blood–placental barriers in the fetus). If the significance of thicknesses of the barriers in signal transduction pathways is some kind of generalized character for every kind of barrier, then these results could provide some sort of the principles for limiting the harmful impacts of NPs’ exposures and offer newer therapeutic paradigms (Sood et al., 2011).

A cellular barrier can avail appreciable protections against nanoparticles’ vulnerabilities and represents the various morphological classes in the bodies of organisms. Examples include the barriers of the corneal epithelium, which in a multilayered combination of tear films can preclude the irritants or allergens and pathogens, from getting entry into the eyes. However, the blood–brain barrier (BBB), which prevents diffusing of microscopical moieties, including bacterial pathogens, into the cerebrospinal fluid (CSF), is composed of a monolayer barrier made up of endothelial cells backed by cells of the astrocytes end feet (Wang and Zhao, 2010; Herrick and Bordoni, 2022). Placental barriers can also govern exchanging of contents between fetal blood and/or tissues and maternal tissues and change in appearance during pregnancy conditions. In the first three months, villi of the placenta get covered up by bilayers of multinucleated cells (syncytio-trophoblast), which directly get rested layers of cells of the cyto-trophoblasts; and then these layers get inter-connected by the gap junctions of connexin-43 channel. In later stages of the pregnancy, placental barriers are majorly, but not solely, made up of a monolayer, with incomplete layers of cells of the cyto-trophoblasts (Keelan, 2011; Woods et al., 2018).

## Consequences of Nanoparticle Toxicity on Fetal Abnormalities

**FIGURE 4:**
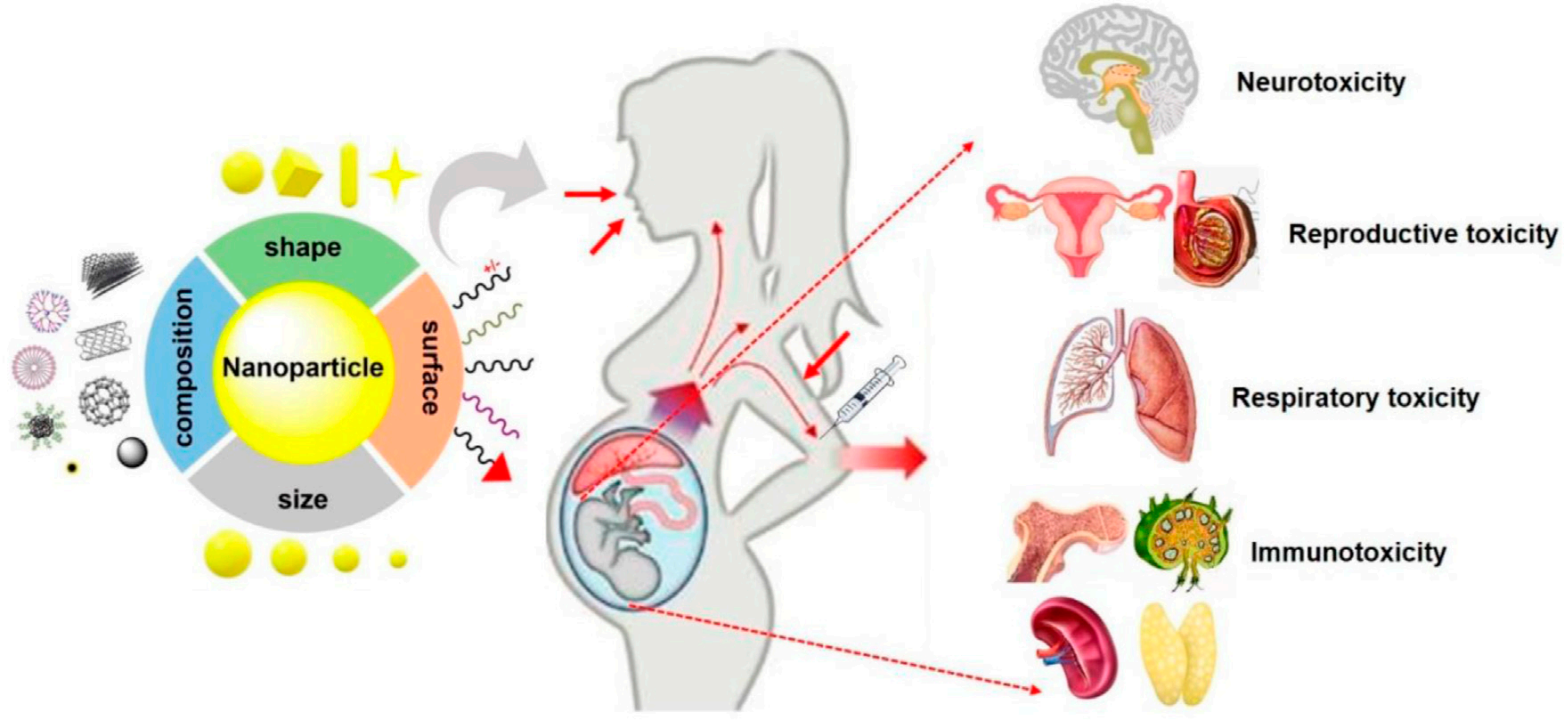
Potential fetal toxicological implications of various types of nanoparticles. Nanoparticle characteristics viz., hydrodynamic diameter (particle) size, surface morphologies, and their composition; other chemistries play significant roles and regulate the fetal toxicity after the maternal exposure to nanoparticles during pregnancy. Furthermore, maternal circumstances and routes of nanoparticles’ exposure critically control the nanoparticle-induced fetal anomalies (reproduced with permission from (Teng et al., 2021).

The potentialities for the exposure to various drug-delivering nanoparticles for causing the developmental toxicities in fetal tissues and that too without any of the direct passages of nanoparticles have earlier been demonstrated, but their mechanisms remain subtle. Nanoparticles have been demonstrated to reduce cell growth, developmental and differentiation capabilities in the hippocampal regions of the developing fetus, could well led to impairments in the memory and learning behavior and change the genetic expressions responsible for the development of the and cause further neuro-behavioral anomalies (Figure 4). These alterations happen due to nanoparticles getting transferring their erosion by-products through the placental barriers into the developing tissues of the fetus. It has also been demonstrated that nanoparticles can restrict the intra-uterine fetal growth and can lead to damages in DNA of fetal tissues that too without the placental barrier trespassing (Sohal et al., 2018). The significance of the placental barrier for developing the brain of the fetus was also previously exhibited. The indirect toxicities of nanoparticles mice models *in vivo*, can culminate in the neuro-developmental anomalies with increased DNA damaging and hippocampal region-reactive astrogliosis in the developing fetus. The researchers could well demonstrate that astrocyte cells behave as the quite vital mediators in neurological toxicities and hippocampal regions in the developing fetus gets specifically affected in the *in vivo* mouse model. Deprivations from food which lead to a stress period can further cause upregulation of the autophagy in placental tissue regions for the protection of the developing brain in the fetus (Broad and Keverne, 2011; Sood et al., 2011; Hawkins et al., 2018).

In another study, the researchers have determined the feasibilities, therapeutic effectiveness and safety, and toxicities of *in utero* gene edition arbitrated by customized multifunctional nanoparticles. It was observed that nanoparticles could get presented to various mouse tissues in the fetus *via* i. v. injections, while they exhibited the most prominent accumulation in the liver tissues of the fetus, which served as the sites for hematopoiesis in the fetus. To this counterpoint, intra amniotic nanoparticle administration resulted in a specific accumulation of nanoparticles in the lungs of the fetus and fetal gut tissues after the gestation period of more than 15 days. It was also observed that both delivery practices were causing invasion to minimal degrees and did not adversely affect the developmental stages of the fetus, its long term surviving capabilities, or reproduction potency (Ricciardi et al., 2018). Applying nanoparticles containing next generation, chemically altered *γ*-PNAs as well as DNA for the *in-utero* administration, the researchers were well able to correct the disease-causing thalassemia mutational anomalies in mouse fetuses for yielding the pertinacious post-natal disease melioration which could be assessed by elevated hemoglobin levels, improvements in morphologies of RBCs (red blood cells), reduced reticulocyte count, and decrease in extra-medullary hematopoiesis, and this happened in combination with clinically pertinent edition criteria in both the adult as well as fetal bone-marrow. Considerably, the researchers noted appreciable and chronic post-natal surviving rewards for *in-utero* administered animal models versus normal control, underlining the possibilities for the translation of this paradigm into clinical settings (Ricciardi et al., 2018; Mitchell et al., 2021).

Fetal tissues have often shown greater sensitivity to environmental hazards in comparison to adults, so it can be claimed that various toxicological compounds in food, drugs, water, and air can lead to complications in pregnancies of humans. It has been estimated that around 1%–3% of women in the United States can undergo sufferings of the recurrent miscarriage and almost 7%–15% of pregnancy conditions get influenced by poor growing conditions of the fetus (called as the intra-uterine growth restrictions. Intra-uterine growth restrictions have the fetus having weights lower than the 10th percentile for their gestational ages and may culminate in the deaths of the fetuses predispose children to prolonged and increased risks of cardiovascular as well as renal disorders. Examinations of the probable risks of nanoparticles leading to miscarriages and intra-uterine growth restrictions have therefore become necessities (Kannan and Vimalkumar, 2021). In various reports the researchers have tried to assess biodistribution and fetal toxicities of surface modified, customized and multifunctional nanoparticles possessing various sizes, including fullerene-based C-60 and titanium dioxide nanoparticles in pregnant mouse models. Their outcomes indicated that nanoparticles having particle size below 100 nm and can lead to induction of the resorption in a growing embryo and could also restrict the growth in the fetus. In addition, it was also noted that modifications of the surfaces of nanoparticles from hydroxides to carboxylic acids or amine-containing functionalities could well lead to the prevention of these complexities of pregnancy conditions. These results also include basic and selective information regarding the probable criteria for the creation, designing and formulating the safe nanoparticles (Mills et al., 1988; Koren et al., 1998; Yamashita et al., 2011).

Biological barriers to *in utero* nanoparticle administrations are actually low in number in comparison to the numbers expected. A nanoparticle-based therapeutic regimen can be directly administered through the umbilical vessels, by amniotic fluid or through specific tissues in the fetus. The restrictions in administrations to the fetus arise from the concern for the interaction of nanoparticles administered to the mother and her fetus. In this regard, viral vectors were successful in delivering the DNA editing systems *in-utero* in the mice models (DeWeerdt, 2018; Massaro et al., 2018; Mitchell et al., 2021). The research was performed for estimation of probable induction of genetic toxicological effects and developmental abnormalities in the fetus, specifically fetal deformities and skeletal anomalies by molybdenum-based NPs administered to the mouse. Orally administered molybdenum nanoparticles lead to a considerable reduction maternal weight, while numbers of fetus, their lengths, and skeletal anomalies mainly comparatively less ossification and less chondroitin tissue formations. The administration of molybdenum-based NPs further leads to induction of the DNA damages and increased the p53 gene expression levels, which performs the crucial part in the maintenance of genetic stabilities and cellular differentiations of both the fetal and maternal fetus tissues. Likewise, the genetic expressions of E–cadherin and N–cadherin, which controls skeletal tissue developments, were increased in female mouse tissues when molybdenum NPs were given to them or to their fetuses. Hence, it was concluded that orally administered molybdenum NPs can induce genotoxic implications and can cause fetal anomalies which necessitate further in-depth research into their probable toxicological outcomes (Mohamed et al., 2020).

In another report, an appreciable enhancement in genetic expressional levels in relevance to innate immunity was seen. Enhancement in toll-like receptor expressions and the downstream pathways (including the Myd88, interleukin-6 and interleukin 1-β) suggested that prenatal exposures to silver-based nanoparticles could change the expression of genes implicated in immunological as well as inflammatory conditions in the brain tissues of the fetus. Enhancement of expression of Hmgb1 is well known to be associated with the damaged-associated molecular patterns and suggested that these silver nanoparticles can well lead to the activation of sterile inflammatory conditions. Overall, it was demonstrated that pre-natal exposure to silver nanoparticles can change the functioning of mitochondria and transcriptional levels of genes which regulate innate immunological responses in the brain tissues of a developing fetus (Amiri et al., 2018).

Furthermore, not everything which gets counter under multifunctional nanoparticles seems evil. Hofmann et al. (2015) tested the synthetic amorphous silica-based nanoparticles for their prenatal toxicities in various doses (viz., 100, 300, or 1,000 mg/kg body weight), studied various parameters including but not limited to corpora lutea numbers, counting of the live and dead fetuses, weights of the fetal and placental tissues, etc. They also assessed the fetuses for any skeletal, external, or visceral anomalies. It was observed that none of the dose levels caused any toxicological implications, and the administration of nanoparticles did not change the c-section parameters and did not affect the weights of the placenta or fetus (Hofmann et al., 2015). Likewise, Wolterbeek and co-workers have assessed the oral toxicity of silica-based nanomaterials in the context of the two-generation reproductive toxicological outcomes in rats. They reported that oral administration of silica-based nanomaterials up to the doses as high as 1,000 mg/kg body weight per day did neither demonstrate any of the adverse impacts on reproduction performances in the rat model nor hampered the growth and development of offsprings in adulthood as seen in two successive progenies (Wolterbeek et al., 2015).

## Regulatory Aspects for Reproductive Toxicity and Safety Assessment of Nanomaterials

Nanoparticles have not only been in the quite progressive states of the research and development but have also initiated their translations into the clinical settings and markets. Industrial and regulatory authorities require some quite coalesced and highly inclusive frameworks and guidelines in terms of the assessments of the reproductive health risk associated with the use of customized and multifunctional nanoparticles applied for drug delivery purposes. Several systems for the identification regarding which types of nanoparticles are susceptible for posing the dangers to individuals’ reproductive health and the environment have been put in place several years ago. However, after various generative decades, where some patchworks of evaluating these reproductive toxicological approaches have been formulated, few of the latest research and developments have been integrated (Hua et al., 2018). These systems of reproductive and regulatory toxicology seem to have fallen numb, much similar to the fairy tale characteristics. In cases of reproductive toxicological paradigms, the main hurdles could have been some of the international regulatory requirements or reproductive toxicological evaluation guidelines. It has thus been more often not significant whether the positive results may represent some of the artifacts (Shu et al., 2013; Kah et al., 2021).

It has, however, become highly relevant for those who can lately require the reproduction of any of the assumed nanoparticle toxicities in organs for validation of the alternate approaches because false-positive outcomes will be quite unmanageable for reproduction when some other alternative tests are performed (Hartung and Sabbioni, 2011). In addition, whether any of the positive outcomes is false can likely remain unnoticed due to the fact that most of the regulatory procedures are performed singly, and also reproductive toxicity reports are generally not disclosed in the public domain. So this self-correction mechanism in the scientific domain may not be in its place, and there has not been any cross-citation among similar reports in various science and research laboratories. It has, therefore, been improbable that scientists can abruptly lead to the production of newer tools, techniques and designs, or newer methodologies possessing greater accuracies and precision (Community Health and Consumer Protection, 2005; Brannen et al., 2016). The solutions to the usage of few animal models and forming comparatively improved anticipations in the middle are the designing of some of the highly integrated reproductive toxicity testing methodologies for the customized and multifunctional nanoparticles. Presently, a distinctive method is the employment of default animal testing subsequently to the application of cell lines and other cell culture techniques and computer-aided designs for defining the modes and mechanism of the reproductive toxicological implications of multifunctional nanoparticles and interpretations as well as further balancing those outcomes. However, the best opportunities for improvements in regulatory implications of reproductive toxicology depend upon tools and techniques with which optimized applications are first undertaken among all the existing informative systems regarding the nanoparticles and other similar nanocarriers, and further these data can be obtained by various other approaches that may not undertake the animal-based testing systems and lead to focused animal-based testing paradigms only when it becomes absolutely essential (Hartung, 2009).

It has now become reasonable to presume that minimum to a five folds greater extent guidelines can be required for accommodating the newer tools techniques and paradigms and tremendously challenging to regulatory authorities and communities. Since the last 2 decades, less than one-tenth of the newer nanoparticles were assessed in these ways, as demonstrated by the appraisals to the European regulatory agencies. Applications of the newer methodologies are impeded or obstructed by traditional and/or obsolete methods on the one hand, while hindrances on the other hand such as the complete absence of harmonized international accords with significant economically viable market regions, viz., Russia, Brazil, and China, are not necessarily accepting newer OECD (Organisation for Economic Co-operation and Development) paradigms. In other scenarios, the conventional animal model– based testing has not been stopped or altered when its alternative approaches were brought in, and hence, the master or pilot testing could still be undertaken to fulfill regulatory or toxicological assessment purposes if necessary and sufficient justifications are furnished. However, when newer or novel approaches cannot fulfill all requirements (these are not suitable for each type of nanoparticles or sometimes are unacceptable by all other member countries), it becomes unmanageable to get rid of the conventional regulatory roadmaps (Hartung, 2009).

Nevertheless, the generalized approaches of DF4-nano groupings are evenly relevant for the reproductive toxicity assessments of nanoparticles *via* almost all the routes of nanoparticle vulnerabilities, like oral, parenteral and/or cutaneous exposures, or localized susceptibility to eye tissues. Normally, nanoparticles possess lower systemic bioavailability after their oral administration and normally do not produce hazardous effects. Also, fewer available reproductive toxicity research reports sometimes do not indicate the nanoparticles’ impacts on the male or female fertilities or developmental stages of the fetus, e.g., silica nanoparticles. Likewise, the presently at-hand *in vitro* as well as *in vivo* reports have not been pointing to the non-intentional permeabilities and systemic bioavailabilities of cutaneously or sub-cutaneously applied nanoparticles, like those nanoparticles employed in sun-screen lotion or creams (Wolterbeek et al., 2015; Landsiedel et al., 2017; Hofmann et al., 2018).

In the contexts of reproductive safety of humans and/or animals’ health and well-being, the European Regulation number 283–2013 and number 284–2013 elucidated the required data for the active pharmaceutical components as well as for plant protection products, that is to be in complete compliance with regulation number 1107–2009. The reproductive toxicity data presently needed for safety evaluation of nanoparticles may include the reports on their pharmacokinetics (both oral and i. v.), acute toxicological outcomes (oral, trans-dermal, inhalational), cutaneous and eyes’ irritability, skin sensitization capabilities, acute toxicities (90 days studies in minimum two species), genotoxic implications (both *in vitro* and *in vivo*), carcinogenic potential, and reproductive toxicities in terms of the other end-points, like neurotoxic potential and immunotoxic reports. Eco-toxicity information needs to be procured by application of proper methodologies, that is, addressing the approximate exposing scenarios. Standardized tests in-hand are required to be applied (viz., *Daphnia magna* (OECD-2012 guidelines), *Folsomia candida* (ISO-2014 regulations), and *Enchytraeus* species (OECD-2004 guidelines), as well as reproduction testing, among others), although some extra-testing may further be suggested, partially because of the increased complexities of circumstances for nanoparticles, which include the specific types of customized multi-functional nanocarriers (Grillo et al., 2021).

Depending largely on products and targeted clinical patient samples, the conduction of reproductive and/or developmental toxicities can further be required for getting conducted parallel to later phases of the clinical studies. Circumstances which can affect the requirements for these analyses may also implicate nanoparticles’ biodistribution to susceptible reproductive organs and tissues and tissue-targeted antigenic expression profiles in the reproductive system (Husain et al., 2015).

## Future Perspectives and Conclusion

As described under the aforementioned headlines, there have been many limitations in present states of expertise for reproductive risk evaluations of multifunctional nanoparticles. Some of the restrictions have been briefly discussed earlier for providing the impulsions to futuristic frameworks in the area of reproductive toxicity of nanoparticles. Various regulatory authorities [viz., EFSA (European Food Safety Authority), OECD etc.] have been actively involved in this arena and have undertaken responsibility for addressing the existing gaps in the assessment of nanoparticles’ reproductive toxicities. For example, newer OECD testing regulations for nanoparticles’ characteristics are currently under development (Rasmussen et al., 2019; Kah et al., 2021). Due to the very diversified nature and characteristics of nanoparticles and the several ways through which humans can get exposed to nanoparticles, a lot of further research needs to be undertaken urgently on reproductive toxicities of nanoparticles, particularly after chronic exposure and exposure during early life.

Of the several nanoparticles described earlier, many of these could induce fetal anomalies and restrict fetal growth in a pregnant mouse model, whereas some others do not initiate these complexities. Some of these nanoparticles could be observed in placental tissues, the brain, and liver of the fetus, while some others could induce anomalies only in their highest doses administered. The hazardous implications observed seen in mouse models could be related to structure- or function-based alterations in placental tissues. Changing the nanoparticles’ surfaces with functional groups like amine or carboxyl may reduce their negative implications, and this can further suggest the significance of surface or interfacial modifications and/or functionalizations. Although some nanoparticles could be chiefly formulated for research- or industry-based applications and may not be for food- or cosmetic-based uses, it can be suggested that potential male and female reproductive toxicities and fetal toxicological implications of these nanoparticles need to be thoroughly investigated with utmost care.

Although nanoparticle-based therapies have revolutionized the ongoing research activities, much remains to be explored about their chronic outcomes and the best pre-clinical animal model systems for studying their responses. As our comprehension of nanoparticles’ pharmacokinetics and pharmacodynamics and their precise mechanism of action continue to advance, several other paradigms that dictate nanoparticle-based therapies and their treatment response will possibly be revealed. This can lead to the creation of further challenges and also newer chances for the development of combinatorial therapies aiming to improve the efficiencies of nanoparticle-based therapies, including multifunctional customized nanoparticles. Adding several other unexplored paradigms to these fields that present additional queries than their answers could well lead to the addition of further volatility. Similarly, nanoparticle scientists can further ask newer questions regarding the effectiveness of these therapies and the ways these NPs interact with reproductive system tissues and developing embryos. By underlining various mechanisms from the non-rigorous lists of features and properties which affect and regulate the outcome of nanoparticle-based medicines, the goal should be the promotion of sentience, encouraging active research and investigation to completely understand the nanoparticles’ effects and their reproductive toxicities and advocating considerations for incorporating these parametric quantities and qualities during earlier stages of experimentation designs.

We have provided many examples of how the methodologies could be realistically employed in practices by giving priority to research on various types of multifunctional nanoparticles. The outcomes have also suggested that optimized applications of research funding can further let in the research into all types of nanoparticles and the experimentation which can minimize the uncertainties in nanoparticles’ shapes and sizes in terms of their hydrodynamic diameters; their solubilities or surface and interfacial reactions often possess the highest capabilities for improvements of hazardous effects at quite lower costs. It always makes proper sense to provide funding to costlier pathophysiological research only when the economic research actions have already been financed. In similarity with other priority-based attempts, the outcomes will always possess some limitations, likely the imprecision in various frameworks, subjectivities of authors’ interpretations of available data, and the smaller numbers of expert opinions regarding the reproductive toxicological implications of customized and multifunctional nanoparticles.
